# Effect of Extending Corticotomy Depth to Trabecular Bone on Accelerating Orthodontic Tooth Movement in Rats

**DOI:** 10.3390/dj10090158

**Published:** 2022-08-29

**Authors:** Thanapat Pathomkulmai, Pannapat Chanmanee, Bancha Samruajbenjakun

**Affiliations:** Orthodontic Section, Department of Preventive Dentistry, Faculty of Dentistry, Prince of Songkla University, Songkhla 90110, Thailand

**Keywords:** corticotomy, osteotomy, depth, accelerated tooth movement

## Abstract

Corticotomy is a surgical procedure that induces injury to the cortical bone to accelerate tooth movement. This study aimed to increase the depth of corticotomy to the trabecular bone and to evaluate the amount and rate of tooth movement and alveolar bone changes in response to various degrees of cortical and trabecular bone injury. Seventy-eight male Wistar rats were randomly divided into four groups based on procedure used: (1) baseline control group of orthodontic tooth movement (OTM) only; (2) OTM + 4 corticotomies (CO); (3) OTM + 4 osteotomies (OS); and (4) OTM + 16 CO. A closed-coil nickel–titanium spring was placed to move the maxillary first molar mesially with a 10 g force. On days 0, 7, 14, and 21, alveolar bone alteration and tooth movement were measured using microcomputed tomography. Significant tooth movement was related to the number and the depth of the perforations. The OTM + 16 CO group showed a greater amount and rate of tooth movement than the OTM + 4 CO group. When osteotomy and corticotomy were compared with the same volume of bone removed, the OTM + 4 OS group had a faster rate of tooth movement than the OTM + 16 CO group during the first week, with significantly reduced bone volume. However, no significant difference was observed in the amount of tooth movement between the OTM + 4 OS and OTM + 16 CO groups at the end of the study. Extending the depth of corticotomy to trabecular bone increased the amount of tooth movement by accelerating the induction and completion of bone remodeling, which accelerated the rate of tooth movement during the initial stage.

## 1. Introduction

Prolonged orthodontic treatment duration leads to some concerning problems that include caries, periodontal disease, and root resorption [1,2,3]. In an effort to reduce treatment duration, surgically assisted orthodontics has gained considerable interest in facilitating orthodontic care. Frost [4] defined the regional acceleratory phenomenon (RAP) as an injury to bone tissue that speeds the healing process. In human long bones, RAP normally begins a few days after damage, peaks between one and two months, and subsides between six and twenty-four months afterwards. This phenomenon is utilized in surgical procedures. In reaction to surgical trauma, bone remodeling is accelerated, which results in transient osteopenia that provides reduced resistance to tooth movement [5,6]. It has been suggested that the intensity of RAP is related to the invasiveness of the injury (i.e., the greater the extent of the injury, the greater the RAP) [4]. Current surgical treatments to accelerate tooth movement range from flapless microperforations to more invasive procedures such as corticotomy and osteotomy [7].

Corticotomy and osteotomy are surgical procedures with varying extents of invasiveness depending on the extent of damage to the cortical and trabecular bone, respectively. In a corticotomy, the trabecular bone is left unaltered and only the cortical bone is cut, drilled, or mechanically changed [5,6]. Previous studies [6,8] have demonstrated that corticotomy-assisted orthodontics allowed teeth to move more quickly than conventional orthodontics. Moreover, decreased root resorption and hyalinization of the periodontal ligament were reported [9]. Corticotomy, however, may result in a one-week period of pain and other postoperative discomfort [10]. The histology details of corticotomy were examined to provide an explanation for the results. Selective alveolar decortication caused a threefold increase in anabolic activity (bone deposition) and catabolic activity (bone degradation) in rats three weeks after corticotomy [11]. Additionally, osteotomy is a surgical procedure that increases the extent of the injury to both the cortical and trabecular bone and quickens tooth movement [12,13,14]. The Le Fort I osteotomy was shown to accelerate alveolar bone remodeling and orthodontic tooth movement in rats [15]. It was also demonstrated that trabecular bone injuries alone could actually accelerate tooth movement. By reducing and removing the interseptal bone of the extraction socket, canine retraction was shown to accelerate [16].

Trabecular (cancellous) bone and cortical bone have different structural characteristics. The outer layer of bone is made up of cortical bone, which is significantly denser than trabecular bone [17,18]. It is more resistant and serves as a significant barrier against tooth movement. As a result, removing some of the cortical bone reduces tooth movement resistance [5,6]. On the other hand, trabecular bone has a larger surface area exposed to bone marrow and blood flow and has a greater turnover rate than cortical bone [19]. Injury to trabecular bone may have a greater effect on accelerating orthodontic tooth movement because bone turnover increases the speed of tooth movement in orthodontics [20], and the distribution of RAP seems to reflect regional vascular structure [4]. Although there is a correlation between increased surgical injury and increased speed of tooth movement, it is not known whether trabecular bone injury or cortical bone injury has the greater effect. In addition to comparing the RAP effects seen in cortical and trabecular bone, this study aimed to evaluate the amount and rate of tooth movement and alveolar bone changes in response to various degrees of cortical and trabecular bone injury.

## 2. Materials and Methods

### 2.1. Sample

**Figure 1:**
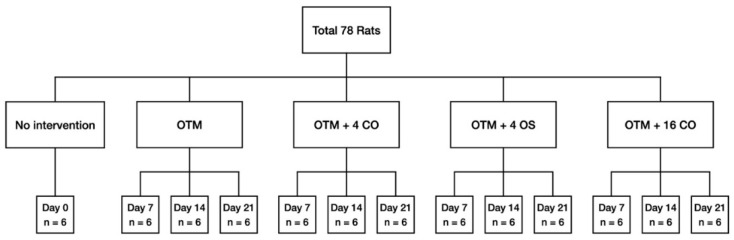
Flow chart representation of the distribution of the animals among the different groups and subgroups with the corresponding observation periods. OTM = orthodontic tooth movement, CO = corticotomies, OS = osteotomies.

In the present study, 78 adult male Wistar rats aged 3–4 months and weighing 300–400 g were used. The animal ethics committee of Prince of Songkla University granted approval for the experimental protocol (MOE 2563-03-038). For at least one week prior to the beginning of the experiment, all rats were kept in housing with controlled lighting and temperature. In the baseline control group (day 0), a total of six rats were used. The remaining 72 rats were randomly assigned into three observation periods of 7, 14, and 21 days. Subsequently, the rats were divided into four groups based on the procedure: (1) orthodontic tooth movement only (OTM); (2) orthodontic tooth movement + 4 corticotomies (OTM + 4 CO); (3) orthodontic tooth movement + 4 osteotomies (OTM + 4 OS); and (4) orthodontic tooth movement + 16 corticotomies (OTM + 16 CO) (Figure 1).

### 2.2. Surgical Procedure

**Figure 2:**
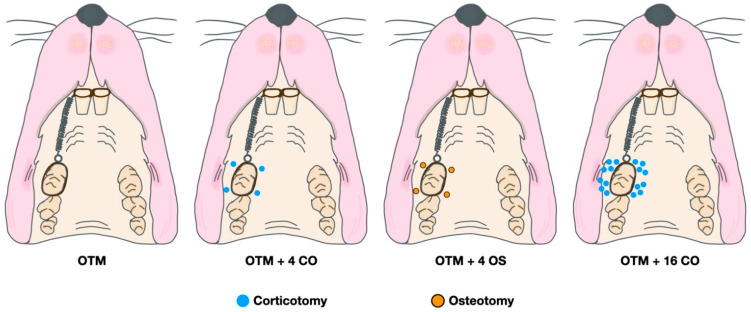
Diagram of surgical and orthodontic tooth movement procedures.

**Figure 3:**
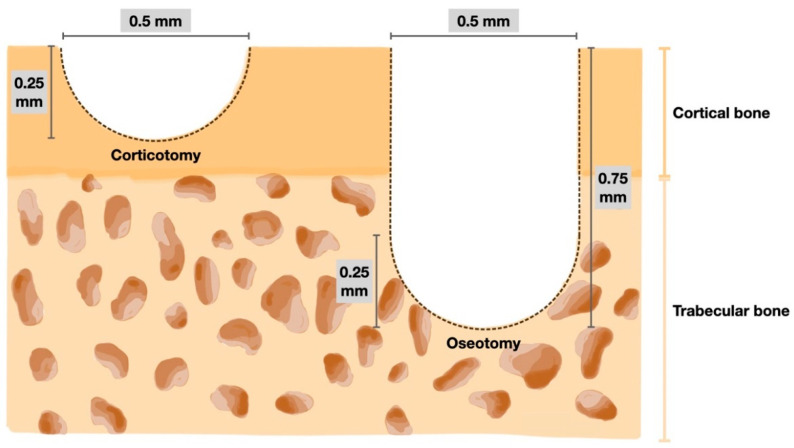
Diagram of the bone perforation for corticotomy (**left**) and osteotomy (**right**).

A random side of the maxilla of each rat was chosen. All operations were performed by the same operator. Prior to the surgical operation and orthodontic appliance placement, the rats were initially sedated with 3% isoflurane (Aerrane, Baxter Healthcare Corporation, Deerfield, IL, USA) and anaesthetized with an intraperitoneal injection mixture of 90 mg/kg of ketamine hydrochloride (Ketajex™, Baxter Pharmaceuticals India Private Ltd., Gujarat, India) and 10 mg/kg of xylazine hydrochloride (X-Lazine, L.B.S. Laboratory Ltd., Bangkok, Thailand). The procedure began with an incision made with a number 11 blade at the gingival sulcus of the mesial line angle of the maxillary second molar. The incision was extended 5 mm mesially along the sulcus of the first maxillary molar to the edentulous ridge. On the buccal and palatal sides, full-thickness periosteal flaps were raised. A slow-speed handpiece equipped with a 0.5 mm round carbide bur was used to perform the corticotomy. Based on the diameter of the burr, each perforation mark was half of the round burr depth, or 0.25 mm deep and 0.5 mm wide. In the OTM + 4 CO group, four perforations were performed: one each at the mesiobuccal, mesiopalatal, distobuccal, and distopalatal aspects of the maxillary first molar. In the OTM + 16 CO group, 16 perforations were made: four points each at the mesiobuccal, mesiopalatal, distobuccal, and distopalatal aspects of the maxillary first molar. Afterwards, 5-0 absorbable material was used to stitch the flaps (Novasorb, Novamedic Co. Ltd., Samutprakan, Thailand). In the OTM + 4 OS group, four osteotomies were performed in the same regions as in the OTM + 4 CO group. The osteotomy perforations were a modified corticotomy in which the depth of the cut was increased to the trabecular bone (0.75 mm). To ensure a uniform depth of 0.75 mm, the bur was marked. Additionally, the volume of bone perforations in the OTM + 4 OS group was approximately equivalent to the OTM + 16 CO group to compare the impact of RAP on cortical and trabecular bone (Figure 2 and Figure 3). Equations (1) and (2) are presented to explain the equivalent volumes of bone removed.

Volume of bone perforation in

OTM + 4 OS group = (π *r*^2^
*h* + 2/3 π *r*^3^) × 4                 = (π (0.25)^2^ (0.5) + 2/3 π (0.25)^3^) × 4         = 0.5238 mm^3^ (1)

Volume of bone perforation in

OTM + 16 CO group = (2/3 π *r*^3^) × 16               = (2/3 π (0.25)^3^) × 16            = 0.5238 mm^3^ (2)

### 2.3. Orthodontic Spring Placement

The ultralight NiTi closed-coil spring (Dentos, Daegu, Korea) was ligated from the maxillary first molar to the central incisors using 0.008″ ligature wires. The spring was activated to produce a 10 g force, which was measured by a force gauge, to move the maxillary first molar mesially using the two maxillary central incisors as anchorage. Light-cure composite resin was applied over the ligature wires to prevent the spring-holding wire from slipping. The rat’s mandibular incisors were trimmed to prevent dislodging of the spring. The spring was closely monitored, and the rats were inspected for signs of pain or stress. The rats were euthanized using a lethal dose of anesthetic drug at each experimental endpoint on days 0, 7, 14, and 21.

### 2.4. Tooth Movement Measurements and Micro-CT Analysis

**Figure 4:**
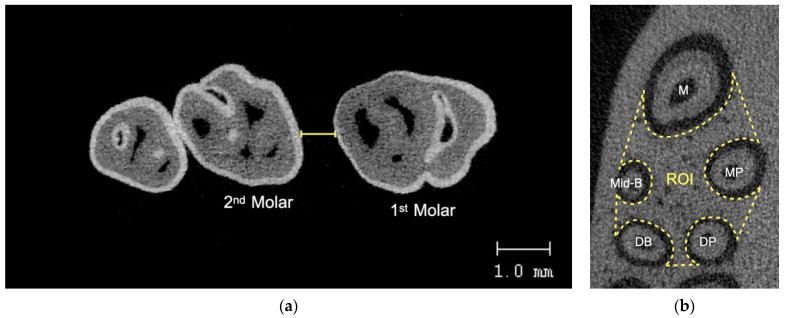
(**a**) Tooth movement measurement using micro-CT, (**b**) ROI for alveolar bone analysis. M = mesial root, Mid-B = mid-buccal, MP = mesiopalatal, DB = distobuccal, DP = distopalatal.

The maxillae of the rats were collected and stored in 10% formalin for one week before scanning using a micro-CT (Scanco µCT 35, Scanco Medical, Bassersdorf, Switzerland) at 70 kVp voltage, 114 µA current, 256 ms exposure time, and 10 µm voxel size. The scanned data were reconstructed using the integrated software. The distance between the most distal point of the first molar crown and the most mesial point of the second molar crown was used to calculate the amount of tooth displacement. By dividing the change in the distance of tooth movement by the distance between time points, the rate (velocity) of tooth movement at each time point was calculated at each week. By calculating the bone volume fraction, which is the ratio of alveolar bone volume to total volume in the area of concern, the alveolar bone modification was evaluated. Serial images were used to evaluate the bone volume fraction in the region of interest (ROI) around the maxillary first molar. The furcation’s most occlusal point and its shortest root’s apex were used to define the ROI vertically, which was usually the mid-buccal root. It was constructed transversely to enclose the interradicular region of the maxillary first molar (Figure 4).

### 2.5. Statistical Analysis

All measurements were done twice at one-month intervals by the same investigator, who was blinded to the treatment groups. Intraobserver reliability was evaluated using the intraclass correlation coefficient. The IBM SPSS^®^ program was used to evaluate the data. The Shapiro–Wilk normality test was used to examine the normality of the data distribution, which was determined to be normal. One-way analysis of variance (ANOVA) was used to compare the differences between groups at each time point. The level of significance was set at *p* < 0.05.

## 3. Results

All rats used in the study remained healthy during the experiment. At the end of the experiment, none of the rats had lost their orthodontic appliances. The intraclass correlation coefficients were 0.985 and 0.951 for the amount of tooth movement and bone volumetric analysis, respectively.

### 3.1. Amount of Tooth Movement

**Figure 5:**
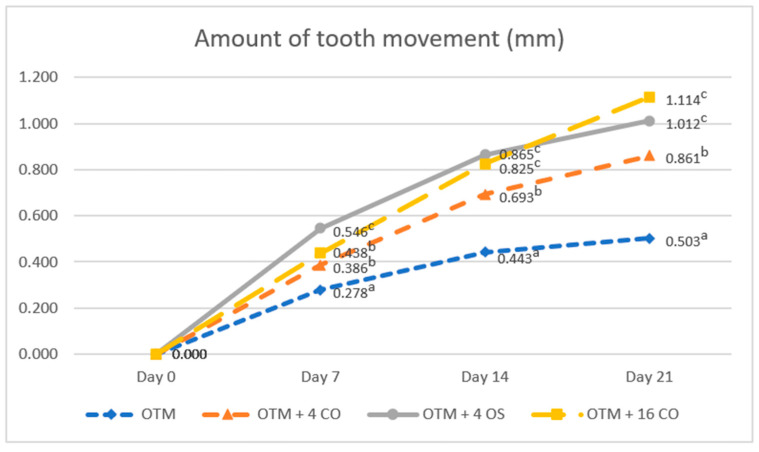
Comparison of the amounts of tooth movement. Different superscript letters (a, b, c) indicate statistically significant differences between groups (*p* < 0.05).

The amount of orthodontic tooth movement was significantly greater in the OTM combined with either the corticotomy or osteotomy alveolar bone surgery groups than in the OTM (control) group for the entire duration of the experiment (OTM group; day 7 = 0.278 ± 0.050 mm, day 14 = 0.443 ± 0.041 mm, day 21 = 0.503 ± 0.046 mm). There was no difference in the amount of tooth movement between the OTM + 16 CO and OTM + 4 CO groups on day 7, but the amount of tooth movement in the OTM + 4 OS group was significantly higher than in the OTM + 16 CO and OTM + 4 CO groups (OTM + 4 CO = 0.386 ± 0.034 mm, OTM + 4 OS = 0.546 ± 0.047 mm, OTM + 16 CO = 0.438 ± 0.049 mm). On day 14, the amount of tooth movement in the OTM + 4 OS group was still higher than in the OTM + 16 CO and OTM + 4 CO groups. However, no significant difference was observed between the OTM + 4 OS and OTM + 16 CO groups (OTM + 4 CO = 0.693 ± 0.040 mm, OTM + 4 OS = 0.865 ± 0.048 mm, OTM + 16 CO = 0.825 ± 0.049 mm). On day 21, the amount of tooth movement was still considerably greater in the OTM + 4 OS and OTM + 16 CO groups than in the OTM + 4 CO group. Although the amount of tooth movement in the OTM + 4 OS group decreased compared to the OTM + 16 CO group, no significant difference was detected between the two groups (OTM + 4 CO = 0.861 ± 0.054 mm, OTM + 4 OS = 1.012 ± 0.080 mm, OTM + 16 CO = 1.114 ± 0.052 mm) (Figure 5).

### 3.2. Rate of Tooth Movement

**Figure 6:**
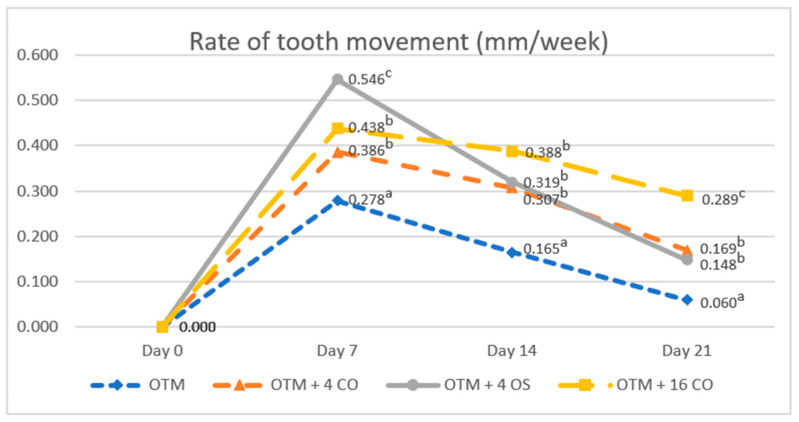
Comparison of the rates of tooth movement. Different superscript letters (a, b, c) indicate statistically significant differences between groups (*p* < 0.05).

The rates of tooth movement in all experimental groups increased in the first week and then continuously decreased in the second and third weeks. The rate of tooth movement in the OTM group was significantly lower than that in the surgically assisted groups throughout the duration of the experiment (OTM group: day 7 = 0.278 ± 0.050 mm/week; day 14 = 0.165 ± 0.078 mm/week; day 21 = 0.060 ± 0.022 mm/week). On day 7, the rate of tooth movement in the OTM + 4 OS group had increased greatly and was significantly higher compared to the OTM + 16 CO and OTM + 4 CO groups. However, no significant difference in the rate of tooth movement was observed between the OTM + 16 CO and OTM + 4 CO groups (OTM + 4 CO = 0.386 ± 0.034 mm/week, OTM + 4 OS = 0.546 ± 0.047 mm/week, OTM + 16 CO = 0.438 ± 0.049 mm/week). On day 14, the rate of tooth movement in the OTM + 4 OS group quickly decreased, being significantly lower than the OTM + 16 CO group but still slightly higher than the OTM + 4 CO group without statistical significance (OTM + 4 CO = 0.307 ± 0.060 mm/week, OTM + 4 OS = 0.319 ± 0.042 mm/week, OTM + 16 CO = 0.388 ± 0.044 mm/week). On day 21, the rate of tooth movement in the OTM + 4 OS group decreased relative to the OTM + 4 CO group. Consequently, there was also no significant difference in the rate of tooth movement between the OTM + 4 OS and OTM + 4 CO groups. However, the rates of tooth movement in these two groups were significantly lower than that in the OTM + 16 CO group (OTM + 4 CO = 0.169 ± 0.058 mm/week, OTM + 4 OS = 0.148 ± 0.050 mm/week, OTM + 16 CO = 0.289 ± 0.042 mm/week) (Figure 6).

### 3.3. Alveolar Bone Changes

**Figure 7:**
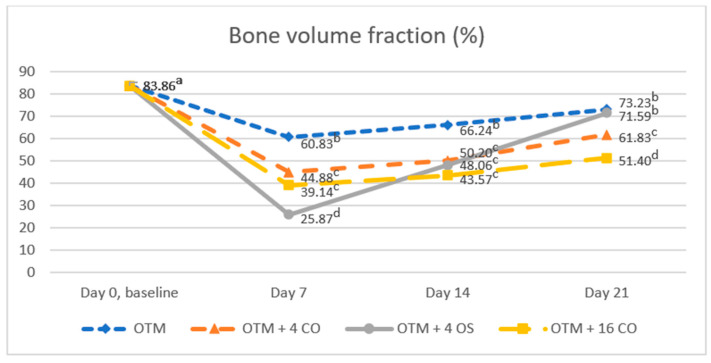
Comparison of the bone volume fraction. Different superscript letters (a, b, c, d) indicate statistically significant differences between groups (*p* < 0.05).

Bone volume fraction values significantly decreased after surgical treatment and tooth movement in all experimental groups compared to the baseline value at all time points. The baseline bone volume fraction value was 83.86 ± 5.29%. On day 7, the bone volume fraction value in the OTM + 4 OS group rapidly decreased and was significantly lower than in the OTM + 16 CO, OTM + 4 CO, and OTM groups. However, on day 7, no significant difference was observed in the bone volume fraction value between the OTM + 16 CO and OTM + 4 CO groups (OTM = 60.83 ± 5.71%, OTM + 4 CO = 44.88 ± 5.72%, OTM + 4 OS = 25.87 ± 8.23%, OTM + 16 CO = 39.14 ± 7.89%). On day 14, the bone volume fraction value increased quickly in the OTM + 4 OS group and also increased gradually in the other groups. As a result, no significant differences in the bone volume fraction values were found among the OTM + 16 CO, OTM + 4 OS, and OTM + 4 CO groups but they were significantly lower than in the OTM group (OTM = 66.24 ± 5.28%, OTM + 4 CO = 50.20 ± 5.19%, OTM + 4 OS = 48.06 ± 5.60%, OTM + 16 CO = 43.57 ± 5.00%). On day 21, the bone volume fraction value in the OTM + 4 OS group continued to increase rapidly to approach the value in the OTM group, and the difference was not statistically significant. Additionally, the bone volume fraction value in the OTM + 16 CO group was significantly lower than in the OTM + 4 CO group, and the value in the OTM + 4 CO group was also significantly lower than in the OTM + 4 OS and OTM groups (OTM = 73.23 ± 4.78%, OTM + 4 CO = 61.83 ± 4.87%, OTM + 4 OS = 71.59 ± 4.64%, OTM + 16 CO = 51.40 ± 5.23%) (Figure 7).

## 4. Discussion

The amount of tooth movement increased significantly as the intensity of alveolar surgery increased. As the number of perforations increased, the amount of tooth movement increased in correlation with the rate and decrease in bone volume fraction. The OTM + 16 CO group showed 29.4% more tooth movement than the OTM + 4 CO group and 121.5% more than OTM group at the end of the study after 21 days of orthodontic force. As perforation depth increased, the OTM + 4 OS group had 17.5% more tooth movement than the OTM + 4 CO group and 101.2% more than the OTM group. According to Frost [4] and Wilcko et al. [21], the severity of bone healing reaction and the subsequent amount and rate of tooth movement are directly proportional to the extent of bone injury. Chang et al. [22] examined the effects of different amounts of alveolar decortication on Wistar rats and found that increasing the amount of alveolar decortication resulted in greater tooth movement. In another study, Abbas et al. [23] compared the effects of corticotomy and piezocision on canine retraction. They discovered that greater invasive corticotomy caused more canine retraction.

Decreased bone volume fraction was likely related to the higher tooth movement rate. The results showed a lower bone volume fraction and a higher rate of tooth movement at all time points in the corticotomy groups and on days 7 and 14 in the osteotomy group compared to the control group. However, there was no significant difference in the bone volume fraction between the osteotomy group and tooth movement only group on day 21, but the rate of tooth movement in the osteotomy group was found to be significantly higher than the tooth movement only group. Thus, a faster tooth movement was not always indicated by a lower bone volume fraction. The decrease in bone volume fraction also revealed the transient osteopenia in RAP, which is a temporary decrease in bone density that happens after bone injury. Normally, bone remodeling maintains a balance between bone deposition and resorption during the repair process. However, in the case of intensive remodeling conditions such as bone surgery, where RAP is induced, bone resorption occurs faster than bone formation. Therefore, bone deposition cannot keep up with the accelerated resorption process, which results in excess bone resorption. When the RAP stimulation subsides, osteoblastic activity returns to normal, new bone fills the remodeling area [24], and osteopenia disappears.

When the effects of RAP in cortical and trabecular bone were compared, the OTM + 16 CO group and the OTM + 4 OS group were clearly distinguishable. Although the same volume of bone was removed from each group, the bone removed was of different types. Only cortical bone was removed in the 16 corticotomies performed in the OTM + 16 CO group, while both cortical bone and trabecular bone were removed from the four osteotomy burr holes in the OTM + 4 OS group. The findings of this study demonstrated that there was no statistically significant difference between the OTM + 16 CO and OTM + 4 OS groups in terms of the amount of tooth movement on days 14 and 21. However, the rate of tooth movement at each time point differed between the two groups. During the first week, the rate of tooth movement in the OTM + 4 OS group was significantly greater than the OTM + 16 CO group. However, during weeks 2 and 3, the rate of tooth movement in the OTM + 4 CO group was significantly less than the OTM + 16 CO group. These results were related to a quick drop in bone volume fraction during the first week after osteotomy, followed by a strong increase during the second week, and subsequently restoration to a value comparable to that of the OTM group in the third week. Results similar to ours were reported by Ji et al. [25], who examined the effects of mandibular osteotomy on tooth movement in rats. They reported a significant decrease in bone volume fraction in tooth movement in the mandibular osteotomy group compared to the tooth movement only group on day 7. However, on day 21 there was no significant difference.

These findings would suggest that osteotomy intensively promoted bone remodeling catabolism during the first week, which led to a decrease in bone volume at an early time. The anabolic responses in bone regeneration were then sharply boosted throughout the second and third weeks. Therefore, the RAP effects in trabecular bone possibly emerged earlier and with a higher intensity than in cortical bone, but they diminished more quickly. A possible explanation is that the cortical bone and trabecular bone are dissimilar in their morphology. Cortical bone is much denser and has higher strength than trabecular bone. Human bone healing in cortical bone requires several steps and several months to complete. In contrast, trabecular bone tissues are less densely packed than cortical bone tissues. In addition, trabecular bone has a large surface area exposed to bone marrow and blood flow that makes it readily accessible to remodeling. Consequently, trabecular bone has a faster turnover rate than cortical bone. Therefore, healing of trabecular bone usually proceeds at a faster rate than cortical bone healing and completes earlier [19,26].

This study showed that osteotomy can be used to increase the RAP effect when the number of corticotomies cannot be increased. Due to the restricted space between the dental roots, it is simpler to extend the depth of corticotomy to trabecular bone than to increase the number of corticotomies. However, the RAP effects on tooth movement and alveolar bone change were different after performing osteotomy and corticotomy in each time period. These findings must be applied with caution to clinical situations due to differences in tissue structure and physiological responses to tooth movement between rats and humans. Therefore, further studies are required to distinguish how the RAP affects cortical and trabecular bone in humans.

## 5. Conclusions

The amount of tooth movement increased significantly as the number or depth of alveolar bone perforations increased. Corticotomy and osteotomy effectively accelerated tooth movement by promoting bone remodeling as evidenced by a reduction in bone volume fraction. Bone remodeling was faster and completed earlier after osteotomy than corticotomy, which resulted in accelerated tooth movement during the early stages of orthodontic treatment.

## Data Availability

Not applicable.
